# Energetics of tryptophan residues in electron transfer and photoprotection of type-II photosynthetic reaction centers

**DOI:** 10.1093/pnasnexus/pgaf278

**Published:** 2025-09-01

**Authors:** Tomoyasu Noji, Keisuke Saito, Hiroshi Ishikita

**Affiliations:** Department of Applied Chemistry, The University of Tokyo, 7-3-1 Hongo, Bunkyo-ku, Tokyo 113-8654, Japan; Research Center for Advanced Science and Technology, The University of Tokyo, 4-6-1 Komaba, Meguro-ku, Tokyo 153-8904, Japan; Department of Applied Chemistry, The University of Tokyo, 7-3-1 Hongo, Bunkyo-ku, Tokyo 113-8654, Japan; Research Center for Advanced Science and Technology, The University of Tokyo, 4-6-1 Komaba, Meguro-ku, Tokyo 153-8904, Japan; Department of Applied Chemistry, The University of Tokyo, 7-3-1 Hongo, Bunkyo-ku, Tokyo 113-8654, Japan; Research Center for Advanced Science and Technology, The University of Tokyo, 4-6-1 Komaba, Meguro-ku, Tokyo 153-8904, Japan

**Keywords:** photoprotection, B-branch electron transfer, ozone layer, evolution, D1 degradation

## Abstract

Tryptophan is the strongest UV chromophore in proteins, and its biosynthesis is the most energy-consuming among all amino acids. In the transmembrane region of purple bacterial photosynthetic reaction centers (PbRC), tryptophan residues are densely concentrated near the inactive electron-transfer branch in subunit M, forming part of the carotenoid binding site. We investigated the redox potentials (*E*_m_) of tryptophan residues in PbRC and O_2_-evolving photosystem II (PSII) by solving the linear Poisson–Boltzmann equation, considering equilibrium with all titratable sites in the entire protein. The tryptophan mediating superexchange electron transfer between the active (bacterio)pheophytin and primary quinone exhibits the highest *E*_m_ value in both PbRC and PSII. In contrast, in PSII, D1-Trp14, oxidized under strong light to trigger the degradation of photodamaged D1 protein, has the lowest *E*_m_ value. In PbRC, a chain of tryptophan residues near the inactive branch forms an *E*_m_ cascade. Quantum mechanical/molecular mechanical calculations suggest that this chain enables electron hole hopping toward the carotenoid, effectively dissipating harmful UV energy. This mechanism likely reflects the photoprotective strategy of PbRC, focusing on UV tolerance rather than oxidative stress.

Significance statement Tryptophan residues play a crucial role in electron transfer and photoprotection in photosynthetic reaction centers. This study suggests that in purple bacterial reaction centers (PbRC), a chain of tryptophan residues forms a redox potential cascade, facilitating electron hole dissipation toward carotenoid to prevent UV-induced damage. In contrast, O_2_-evolving photosystem II (PSII) evolved mechanisms to manage oxidative stress in oxygen-rich environments. These divergent photoprotective strategies reflect adaptations to the distinct environmental pressures faced by early life on Earth, with PbRC focusing on UV tolerance and PSII evolving mechanisms to cope with reactive oxygen species. This research enhances our understanding of the structural and functional design of photosynthetic proteins in response to their environments.

## Introduction

Charge separation and photoprotection are intrinsically linked processes that together sustain efficient photosynthesis in photosynthetic reaction centers (1–5). Type-II reaction centers, such as photosynthetic reaction centers from purple bacteria (PbRC) and photosystem II (PSII), are characterized by the presence of quinone as a terminal electron acceptor. In *Rhodobacter sphaeroides* PbRC, following the electronic excitation of the bacteriochlorophyll pair (P_A_ and P_B_) at 860 nm, electron transfer proceeds via accessory bacteriochlorophyll (B_A_), bacteriopheophytin (H_A_), the primary quinone (Q_A_) along the transmembrane axis in the A-branch, and finally to the secondary quinone (Q_B_) in the B-branch. In PSII, the structurally corresponding cofactors are the chlorophyll pair (P_D1_ and P_D2_), accessory chlorophyll (Chl_D1_), pheophytin (Pheo_D1_), Q_A_ in the D1-branch, and Q_B_ in the D2-branch. However, the initial electron donor is Chl_D1_, due to the weak electronic coupling between P_D1_ and P_D2_ and the presence of the Mn_4_CaO_5_ water-splitting cluster and associated proton-transfer pathways (6).

In type-II reaction centers, electron transfer occurs predominantly along one of the two symmetrically arranged electron transfer chains. While the overall cofactor arrangement is symmetrical, the cofactors themselves differ in functional properties between the two branches (7). This asymmetry is primarily due to the higher *E*_m_ values of chlorophyll cofactors in the active electron-transfer branch, which stabilizes the resulting anionic state relative to the inactive branch (6, 8). Differences in electronic coupling between cofactors further contribute to this asymmetry (9). In particular, the electronic coupling between H_A_/Pheo_D1_ and Q_A_ is larger than that between H_B_/Pheo_D2_ and Q_B_ due to the presence of Trp-M252/D2-Trp253 in the active branch. These tryptophan residues facilitate superexchange electron transfer more effectively than phenylalanine residues in the inactive branch (10). Fine-tuning these factors could potentially enhance electron transfer along the inactive branch. Indeed, in PbRC, mutating the P_B_ ligand His-M202 (His-M200 in *Blastochloris viridis* PbRC) with leucine, resulting in the formation of a bacteriochlorophyll/bacteriopheophytin heterodimer special pair, or swapping the Tyr-M210/Phe-L181 pair near B_A_/B_B_ to phenylalanine/tyrosine, have been investigated in this context (11–14). While such modifications can enhance initial charge separation via B_B_^•−^ in the B-branch, studies indicate that electron transfer along the B-branch remains limited, even with additional mutations designed to alter electron-transfer asymmetry. Specifically, electron transfer to the B-branch is only a few percent (∼6%) when compared to wild-type PbRC (12, 13).

The efficiency of charge separation is critical not only for promoting forward electron transfer but also for inhibiting backward electron transfer, i.e. charge recombination. For O_2_-evolving PSII, preventing charge recombination is essential for survival, as recombination at Chl_D1_ produces triplet chlorophyll, which generates harmful singlet O_2_ (^1^O_2_). To avoid such risks, PSII employs several photoprotective mechanisms. On the electron acceptor side, PSII increases the redox potential (*E*_m_) of Q_A_, thereby enlarging the *E*_m_ gap between Q_A_ and Pheo_D1_, which helps prevent backward electron transfer (2, 15). Once water splitting is impaired and the positive charge (electron hole) remains on the reaction center chlorophyll, *β*-carotene facilitates hole transfer (hole hopping) to the heme protein subunit, cytochrome *b*_559_ (3, 16–18). Furthermore, an already photodamaged D1 protein, which occurs frequently due to the presence of Mn_4_CaO_5_, is degraded by the FtsH protease and substituted with an intact one (D1 turnover). Recent studies suggested that the oxidation of a tryptophan residue in the D1 protein may serve as a trigger for D1 degradation (19). Although to a lesser extent, the D2 protein is also degraded and replaced when photodamaged (20, 21).

Since PbRC does not evolve O_2_, it does not require all the photoprotective mechanisms observed in PSII. However, as PbRC also undergoes charge separation during photosynthesis, photoprotective strategies are still essential for its sustainable and efficient function. In fact, in *Rhodobacter sphaeroides* strain R26, which lacks carotenoid spheroidene near B_B_, UV-B irradiation inhibits charge separation and results in the loss of the reaction center protein subunits through the formation of ^1^O_2_ (22). This photodamage is prevented in the wild-type PbRC, where spheroidene quenches the ^3^[P_A_P_B_] triplet state via B_B_ (23). In addition, it has been reported that PbRC is less sensitive to UV-B irradiation than PSII, likely due to the absence of the UV-B sensitive oxygen-evolving complex (24). Nevertheless, chlorin cofactors, such as bacteriochlorophylls, absorb strongly in the UV-A (320 to 400 nm) and UV-B (280 to 320 nm) regions (N, L, and M bands (25)) (26), potentially making PbRC vulnerable to UV-induced damage.

**Fig. 1.**
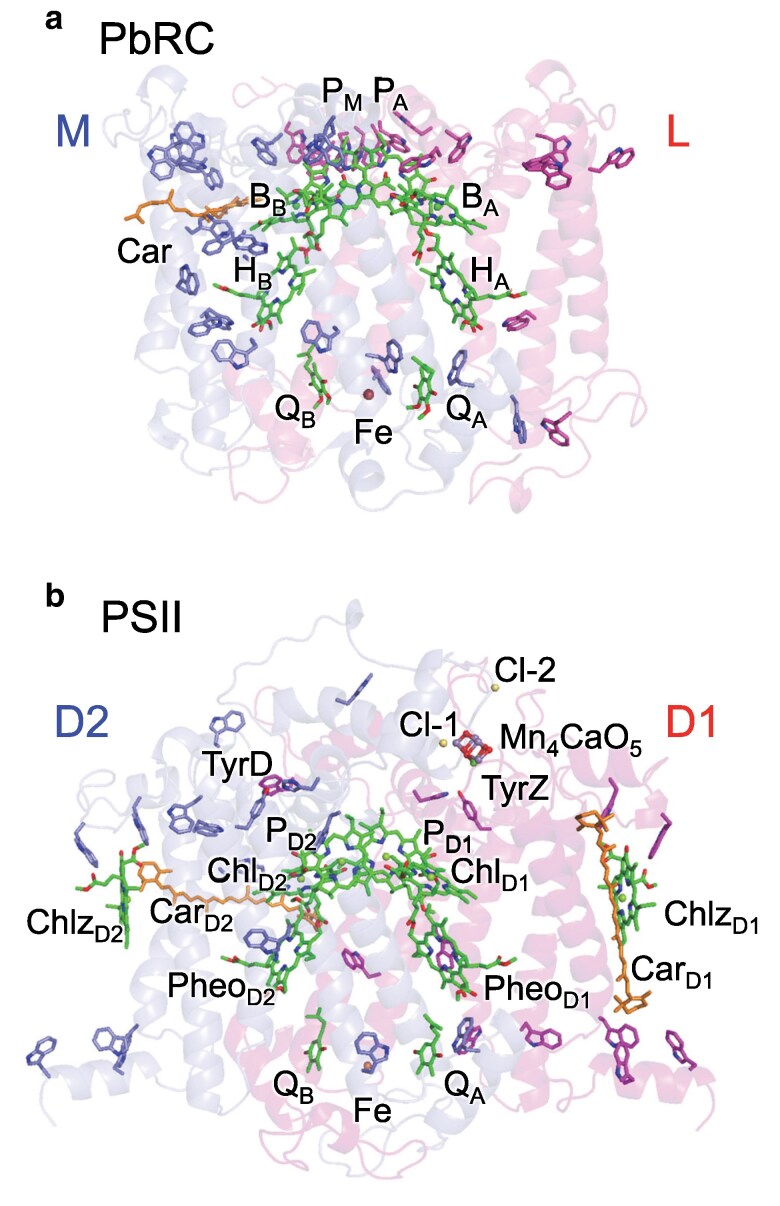
Distribution of tryptophan residues in type-II reaction centers. a) Subunits L and M of *Rhodobacter sphaeroides* PbRC. b) D1 and D2 proteins of PSII from *Thermosynechococcus vulcanus*. Subunit H in PbRC is not shown for clarity. Tryptophan residues are shown in stick representation, highlighting their spatial distribution.

A unique feature of the spheroidene binding site is the presence of a cluster of tryptophan residues at van der Waals contact distances: Trp-M66, M75, M115, M157, and M171 (27). Remarkably, in the transmembrane region of *Rhodobacter sphaeroides* PbRC, tryptophan residues are highly concentrated near the inactive B-branch in subunit M, whereas only one tryptophan residue is present in subunit L (Fig. 1). Given that tryptophan biosynthesis is the most energy-consuming pathway among all amino acids, its presence is restricted to specific regions where its unique physicochemical properties are indispensable (28). The indole side-chain of tryptophan, with an absorption maximum at 280 nm, is the strongest UV chromophore in proteins (29), and its sacrificial absorption of UV light effectively shields the protein from UV-induced damage (30). Indeed, the biological importance of tryptophan in UV-B sensing has been demonstrated in the plant photoreceptor UVR8. It employs a cluster of tryptophan residues, whose UV-B-induced conformational change initiates downstream signaling pathways (31). This highlights the functional versatility of tryptophan as a standalone UV-B chromophore and supports the scenario that localized tryptophan clusters in PbRC may likewise serve specialized roles beyond general UV absorption.

In PbRC, approximately half of the electronically excited tryptophan residues are quenched by the reaction center cofactors within 70 ps (32), suggesting efficient energy transfer from tryptophan to these cofactors. However, if the primary goal were merely to prevent UV damage, one might question why tryptophan residues are densely positioned in the specific region of the protein, despite the availability of other UV-absorbing residues such as tyrosine and phenylalanine, which are less costly to biosynthesize. This presents an apparent paradox: nature appears to selectively invest in such a biosynthetically expensive amino acid rather than minimizing its usage.

Intriguingly, Gray and Winkler reported that approximately one-third of all proteins contain chains of tryptophan or tyrosine residues composed of more than three members, which in some cases can transfer potentially damaging oxidizing equivalents (i.e. electron holes) away from sensitive active sites toward the protein surface (33). Supporting this redox-protective role, about half of O_2_-using oxidoreductases possess tryptophan or tyrosine chains longer than three residues (33). However, despite the presence of tryptophan clusters in PbRC, the detailed energetics of these residues, apart from specific sites (e.g. Trp-M252 (10)), remain unexplored.

Here, we investigate the *E*_m_ values of tryptophan residues in the reaction centers of PbRC and PSII by solving the linear Poisson–Boltzmann (PB) equation, taking into account the protonation states of all titratable sites in the entire protein. Based on the calculated *E*_m_ values, we further examine their detailed energetics, using a quantum mechanical/molecular mechanical (QM/MM) approach in the presence of the entire protein environment.

## Results

**Fig. 2.**
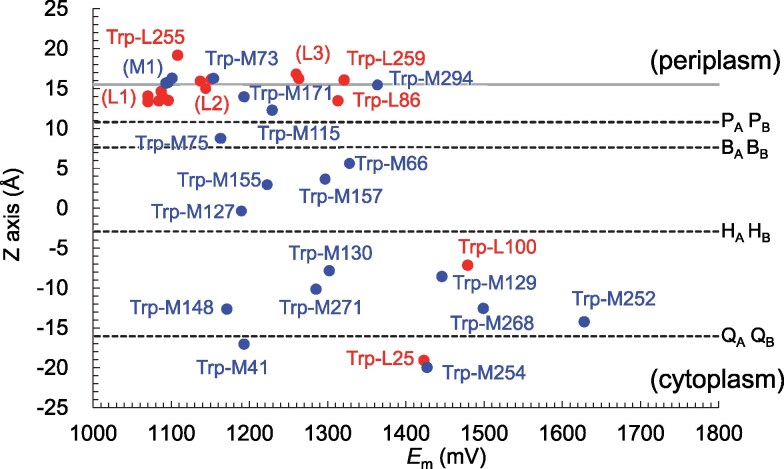
*E*_m_(Trp/Trp^•**+**^) values in subunits L and M of the PbRC structure. The transmembrane Z-axis is defined based on the *Orientations of Proteins in Membranes* (OPM) database (34). The gray horizontal line on the periplasm side represents the interface between the transmembrane and bulk regions, as defined based on the OPM database. The corresponding interface on the cytoplasm side aligns closely with the positions of quinones and is not explicitly shown for clarity. Closed circles indicate *E*_m_ values in subunits L and M. Dotted horizontal lines represent the positions of cofactors along the transmembrane Z-axis. (L1): Trp-L262, Trp-L59, Trp-L265, Trp-L151, Trp-L271, and Trp-L266; (L2): Trp-L272, Trp-L51, and Trp-L263; (L3): Trp-L156 and Trp-L142; (M1): Trp-M80, Trp-M185, and Trp-M297 (listed in decreasing order along the Z-axis for each group).

**Fig. 3.**
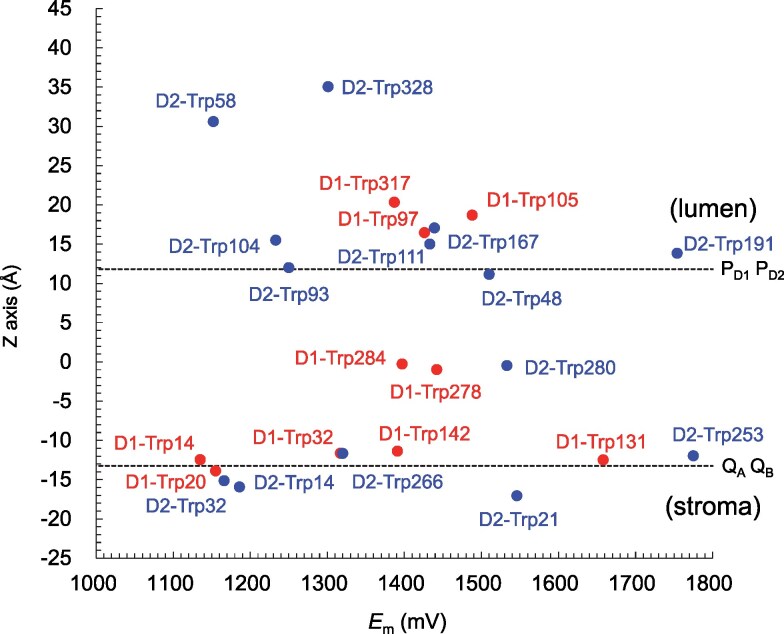
*E*_m_(Trp/Trp^•**+**^) values in D1 and D2 of the PSII structure. The transmembrane Z-axis is defined based on the OPM database (34). The gray horizontal line on the periplasm side represents the interface between the transmembrane and bulk regions, as defined based on the OPM database. The corresponding interface on the cytoplasm side aligns closely with the positions of quinones and is not explicitly shown for clarity. Closed circles indicate *E*_m_ values in D1 and D2 proteins. Black dotted horizontal lines represent the positions of cofactors along the transmembrane Z-axis.

In the PbRC structure, calculated *E*_m_(Trp/Trp^•**+**^) values range from 1,070 to 1,630 mV (Fig. 2). In the region near the periplasmic bulk surface on the P_A_ and P_B_ side, these values fall between 1,070 and 1,370 mV. Near the cytoplasmic bulk surface on the Q_A_ and Q_B_ side, the values range from 1,180 to 1,430 mV. In contrast, these *E*_m_ values shift upward to between 1,160 and 1,630 mV in the transmembrane region. A similar trend is observed in PSII (Fig. 3), where the *E*_m_(Trp/Trp^•**+**^) values are higher in the transmembrane region compared to the lumenal and stromal bulk surfaces.

**Fig. 4.**
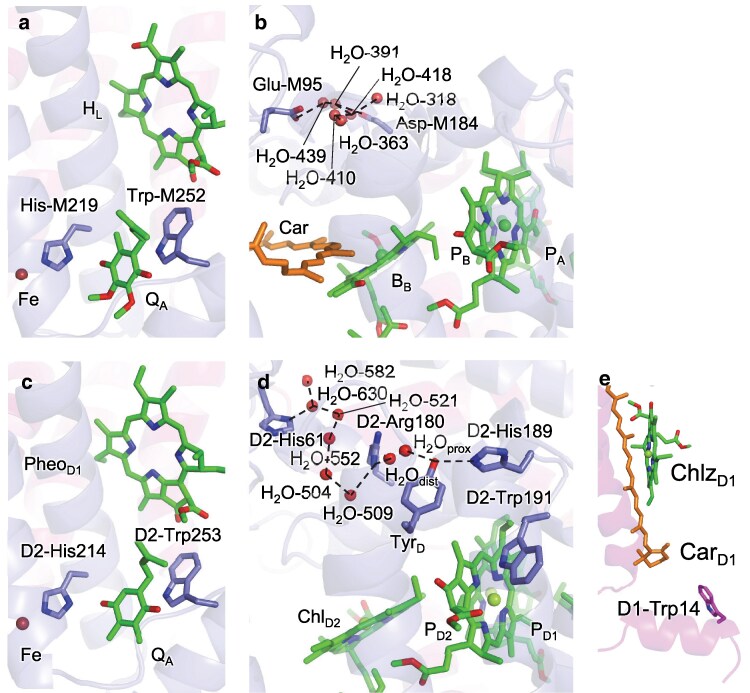
Protein environments for characteristic tryptophan residues. a) Trp-M252 in PbRC, b) H-bond network near the bacteriochlorophyll pair (P_A_ and P_B_) in PbRC. A tryptophan residue corresponding to D2-Trp191 is not conserved in PbRC. c) D2-Trp253 in PSII. d) H-bond network near the chlorophyll pair (P_D1_ and P_D2_) and D2-Trp191 in PSII. H_2_O_prox_ and H_2_O_dist_ represent water molecules proximal and distal to TyrD, respectively (35). e) D2-Trp14 in PSII. Dotted lines indicate H-bonds of water molecules.

Among all tryptophan residues in the PbRC structure, Trp-M252 (Fig. 4a), located near H_A_ and Q_A_, exhibits the highest *E*_m_ value (1,630 mV, Fig. 2). Similarly, D2-Trp253 (Fig. 4c), the corresponding conserved residue in PSII, also shows the highest *E*_m_ value among all tryptophan residues in PSII (1,780 mV, Fig. 3). These residues have been proposed to mediate the superexchange electron transfer from H_A_ to Q_A_ in PbRC (36, 37) and from Pheo_D1_ to Q_A_ in PSII (10).

The *E*_m_ values of tryptophan residues may reflect the energy level of the intermediate virtual state in superexchange when the Trp^•+^-like intermediate state is the virtual state. However, the high *E*_m_ values themselves are not necessarily essential for efficient superexchange electron transfer. In fact, D2-Trp253 facilitates superexchange electron transfer via unoccupied molecular orbitals (via the [Trp]^•−^-like intermediate state) rather than via occupied molecular orbitals (i.e. via the [Trp]^•+^-like intermediate state) (10).

**Fig. 5.**
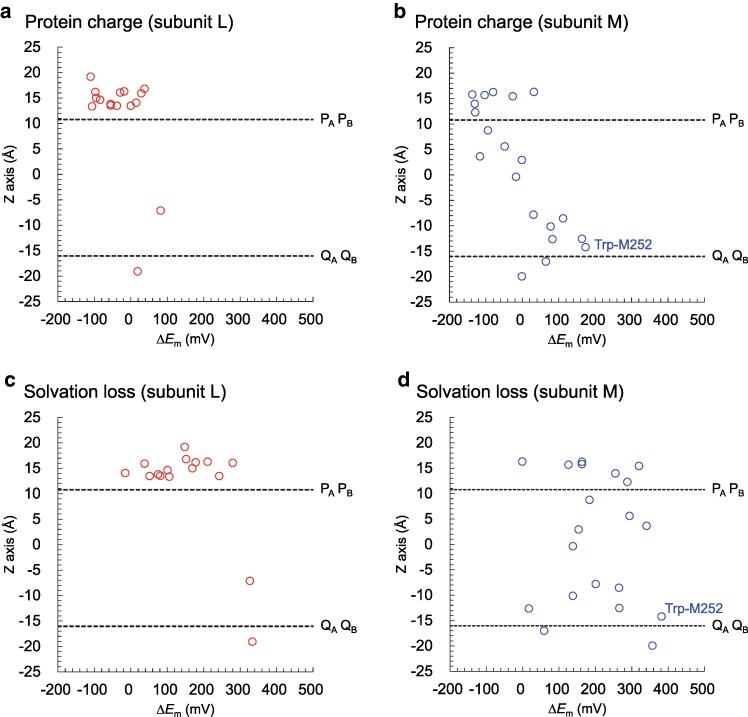
Contributions of the PbRC protein environment to shifts in *E*_m_(Trp/Trp^•**+**^) (Δ*E*_m_). a) Contributions of protein charges to shifts in *E*_m_(Trp/Trp^•**+**^) in tryptophan residues of subunit L. b) Contributions of protein charges to shifts in *E*_m_(Trp/Trp^•**+**^) in tryptophan residues of subunit M. c) Contributions of solvation loss to shifts in *E*_m_(Trp/Trp^•**+**^) in tryptophan residues of subunit L. d) Contributions of solvation loss to shifts in *E*_m_(Trp/Trp^•**+**^) in tryptophan residues of subunit M. Open circles indicate shifts in *E*_m_(Trp/Trp^•**+**^) for tryptophan residues of subunits L and M. Dotted horizontal lines represent the locations of P_A_P_B_ and quinones, i.e. the transmembrane region.

The upshifted *E*_m_(Trp/Trp^•**+**^) values for these conserved tryptophan are due to their highly packed protein environment near H_A_/Pheo_D1_ and Q_A_, which restricts access to water molecules and destabilizes the charged redox state (Fig. 5d). It should be noted that, tryptophan residues located in solvent-exposed regions experience greater stabilization of their oxidized state through electrostatic interactions with water molecules, resulting in lower *E*_m_ values. In contrast, tryptophan residues buried in a highly packed protein environment (e.g. the transmembrane region) are shielded from bulk water, reducing this stabilization. This phenomenon, known as “solvation loss” in the protein environment, leads to higher *E*_m_ values.

**Fig. 6.**
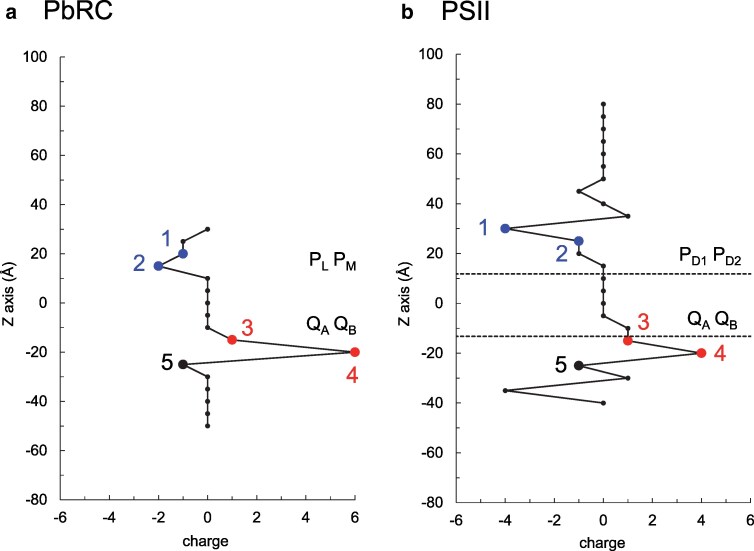
Localization of negative and positive charges along the transmembrane axis. a) Protein charges in subunits L and M in PbRC. Regions 1 and 2 are the two adjacent layers (each 5 Å thick) from the periplasmic protein surface, and regions 3 and 4 are the two adjacent layers (each 5 Å thick) from the cytoplasmic protein surface. The details of these regions are as follows: Region 1: +3 from positively charged groups (Lys-L268, Arg-M87, and Lys-M110) and −4 from negatively charged groups (Asp-L257, Asp-L261, Asp-M292, and Glu-M111). Region 2: +3 from positively charged groups (Arg-L135, Arg-M164, and His-M301) and −5 from negatively charged groups (Asp-L155, Asp-M88, Glu-M95, Glu-M173, and Asp-M184). Region 3: +2 from positively charged groups (Arg-L103 and Arg-M132) and −1 from a negatively charged group (Glu-L106). Region 4: +8 from positively charged groups (Arg-L109, Arg-L231, Arg-M136, Lys-M144, Arg-M253, Arg-M267, and Fe^2+^) and −2 from negatively charged groups (Glu-M2 and Glu-M234). Region 5: +5 from positively charged groups (Lys-L202, Lys-L204, Arg-L207, Arg-M241, and Arg-M247) and −6 from negatively charged groups (Asp-L210, Glu-L212, Asp-M17, Glu-M22, Asp-M23, and Glu-M246). (b) Protein charges in D1 and D2 proteins in PSII. Regions 1 and 2 are the two adjacent layers (each 5 Å thick) from the lumenal protein surface, and regions 3 and 4 are the two adjacent layers (each 5 Å thick) from the stromal protein surface. The details of these regions are as follows: Region 1: +4 from positively charged groups (D1-His304, D1-Arg323, D1-Arg334, and D1-His337) and −8 from negatively charged groups (D1-Asp59, D1-Asp308, D1-Glu329, D2-Glu307, D2-Asp308, D2-Glu312, D2-Glu323, and D2-Glu337). Region 2: +20 from positively charged groups (D2-Arg103, D2-Arg304, D2-Lys317, D2-Arg326, and Mn(III)_2_Mn(IV)_2_Ca^2+^ of Mn_4_CaO_5_) and −21 from negatively charged groups (D1-Asp61, D1-Asp103, D1-Glu104, D1-Glu189, D1-Asp319, D1-Glu333, D1-Asp342, D2-Asp297, D2-Glu302, D2-Asp333, O_5_ of Mn_4_CaO_5_, and Cl^−^). Region 3: +3 from positively charged groups (D1-Arg27, D1-Arg136, and D2-Arg128) and −2 from negatively charged groups (D1-Glu132 and D2-Glu131). Region 4: +7 from positively charged groups (D1-Arg16, D1-Arg140, D2-Lys23, D2-Arg26, D2-Arg139, and Fe^2+^) and −3 from negatively charged groups (D2-Asp19, D2-Glu219, and HCO_3_^−^). Region 5: +8 from positively charged groups (D1-Arg257, D1-Arg269, D2-Arg24, D2-Arg134, D2-Arg233, D2-Arg251, D2-Lys264, and D2-Arg265) and −9 from negatively charged groups (D1-Glu15, D1-Asp25, D1-Glu229, D1-Glu231, D1-Glu244, D2-Asp16, D2-Asp20, D2-Asp25, and D2-Glu242). The sign of each charged group is based on the calculated protonation state, and each charge is approximated to a unit charge for simplicity.

In this low-dielectric protein environment, the influences of protein charges on *E*_m_(Trp/Trp^•**+**^) are particularly pronounced (Figs. 5b and S1b). Although no charged residues are present near Q_A_, unlike the protein environment near Q_B_, there is a greater accumulation of positive charges on the cytoplasmic side (e.g. Arg-M241, Arg-M247, and Arg-M253 in the A-branch, located >10 Å from Q_A_) than on the periplasmic side near P_A_P_B_ (Fig. 6). This accumulation of charges with opposite signs on the periplasmic and cytoplasmic sides, coupled with the low-dielectric nature of the hydrophobic protein environment around Q_A_, results in the significant upshift in the *E*_m_(Trp/Trp^•+^) values for Trp-M252 and D2-Trp253.

In PSII, D2-Trp191 similarly exhibits the high *E*_m_(Trp/Trp^•**+**^) value (Fig. 3). D2-Trp191 is positioned nearly parallel to the axial ligand of P_D2_, D2-His197, near the lumenal region (Fig. 4d). Its indole plane is perpendicular to and van der Waals contact with the P_D2_ chlorin ring. Consequently, while the P_D1_ chlorin ring exhibits a “doming” out-of-plane distortion frequently seen in histidine-ligated chlorophylls, the P_D2_ chlorin ring uniquely exhibits a “saddling” out-of-plane distortion due to the presence of D2-Trp191 (38). This tightly packed protein environment destabilizes its charged redox state, thereby increasing *E*_m_(Trp/Trp^•**+**^) (Fig. S1d). In addition, the presence of positive charges on the D2 side (e.g. D2-His61 and D2-Arg180) compared to the D1 side (e.g. D1-Asp61 and Cl^−^ at D1-Asn181) not only increases *E*_m_(P_D2_) relative to *E*_m_(P_D1_) (8, 39) (resulting in P_D1_^•**+**^ > P_D2_^•**+**^ (40–42)) but also increases *E*_m_(Trp/Trp^•**+**^) of D2-Trp191 (Fig. S1b). Notably, D2-His61 and D2-Arg180 on the D2 side are involved in the proton releasing pathway from redox-active D2-Tyr160 (TyrD-OH) and facilitate the formation of the neutral radical TyrD-O^•^ (35, 43), whereas D1-Asp61 and Cl^−^ at D1-Asn181 on the D1 side are involved in the H-bond network for the release of the proton from substrate water molecules at the Mn_4_CaO_5_ cluster (44–49) (Fig. 4d). Since these proton-transfer events are absent in PbRC, the high *E*_m_(Trp/Trp^•**+**^) value of D2-Trp191 is a specific characteristic of PSII.

In contrast to the positively charged residues D2-His61 and D2-Arg180, which increase the *E*_m_(Trp/Trp^•**+**^) value of D2-Trp191 in the lumenal region of PSII, protein charges in the periplasmic region of PbRC lead to decreases in *E*_m_(Trp/Trp^•**+**^) (Fig. 5a, b). This difference primarily arises from the subunit assembly variations between the two type-II reaction centers. Basic residues such as D2-His61 and D2-Arg180 in PSII, which are involved in the proton-transfer pathway, are replaced with acidic residues such as Glu-M95 and Asp-M184 in PbRC (Fig. 4b). In PbRC, Glu-M95 and Asp-M184 are essential for forming salt-bridge with basic residues of the membrane extrinsic protein, cytochrome *c*_2_ (50). These acidic residues also decrease *E*_m_(B_B_) with respect to *E*_m_(B_A_) and facilitate A-branch electron transfer via B_A_ (8). This fundamental difference between the presence of acidic residues (Glu-M95 and Asp-M184) in PbRC and basic residues (D2-His61 and D2-Arg180) in PSII explains why protein charges tend to lower *E*_m_(Trp/Trp^•**+**^) for the majority of tryptophan residues in the periplasmic region of PbRC (Fig. 5b) compared to the lumenal region of PSII (Fig. S1b).

Among all tryptophan residues in the PSII structure, D1-Trp14, located on the stromal protein surface (Fig. 4e), exhibits the lowest *E*_m_(Trp/Trp^•**+**^) value, indicating that it is the most easily oxidizable tryptophan (1140 mV, Fig. 3). D1-Trp14 has been implicated in the photoprotective mechanism of PSII (19). Under high-light conditions, the D1 protein, which serves as the primary binding site for the O_2_-evolving Mn_4_CaO_5_ cluster, can be photodamaged. When this happens, the damaged D1 protein is degraded by the FtsH protease and replaced with an intact D1 protein, thereby restoring PSII functionality. Oxidative post-translational modification of tryptophan in PSII is a key step that triggers the degradation of the damaged D1 protein by the FtsH protease. Intriguingly, oxidation of D1-Trp14 has been unambiguously observed in *Arabidopsis* PSII under high-light conditions, potentially serving as a signal for D1 degradation (19). The low *E*_m_(Trp/Trp^•**+**^) value of D1-Trp14 is due to its high exposure to bulk water, as indicated by the minimal contribution of solvation loss to *E*_m_(Trp/Trp^•**+**^) (Fig. S1c). The low *E*_m_(Trp/Trp^•**+**^) value for D1-Trp14 is consistent with its susceptibility to oxidation, and its exposure on the stromal protein surface correlates with its high reactivity with ^1^O_2_. The absence of a corresponding tryptophan residue in PbRC may reflect the lack of the O_2_-evolving capability and the use of alternative photoprotective mechanisms.

## Discussion

**Fig. 7.**
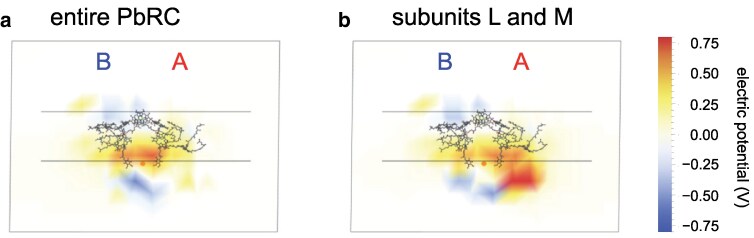
Electric field generated by protein charges in PbRC. a) Electric field generated by the entire PbRC protein. b) Electric field generated by the reaction center protein subunits, L and M. For simplicity, the electric field is shown only on a single sliced plane along the pseudo-*C*_2_ axis. While this depiction represents the electric field exclusively within this plane and does not include the cumulative contributions from all planes along the axis perpendicular to the paper, it serves as a representative visualization of the electric field along other such planes. The gray horizontal lines represent the interface between the transmembrane and bulk regions, as defined based on the OPM database (34). Labels A and B denote the A- and B-branches, respectively. The electric field direction is indicated by the transition from positive potential regions to negative potential regions, corresponding to the same direction as hole hopping and the opposite direction to electron transfer.

The *E*_m_ values of tryptophan residues in the reaction center proteins of PbRC reveal that in the transmembrane region, the contributions of protein charges to *E*_m_(Trp/Trp^•**+**^) increase toward the cytoplasmic side (Fig. 5b). Consistent with this finding, the present analysis further indicates the presence of an electric field generated by protein charges (Fig. 6), which facilitates electron transfer toward Q_A_ (Fig. 7a).

The protein charges in the transmembrane intrinsic region (i.e. within the membrane) stabilize an electron more effectively in the B-branch region than in the A-branch region (Fig. S2a). In contrast, the protein charges in the transmembrane extrinsic region (i.e. outside the membrane), particularly the negatively charged cytochrome *c*_2_ binding interface formed by residues such as Glu-M95 and Asp-M184, destabilize an electron in the periplasmic B-branch region (8, 50) (Fig. S2b). Specifically, subunit L provides positive charges in the cytoplasmic A-branch region (Fig. S2c), while subunit M provides negative charges in the periplasmic B-branch region (Fig. S2d). Together, these features promote electron transfer along the A-branch toward Q_A_, perpendicular to the membrane plane, enhancing A-branch electron transfer relative to the B-branch (Fig. 7b). In contrast, subunit H generates an electric field that facilitates electron transfer from Q_A_ to Q_B_ in the direction parallel to the membrane plane (Fig. S2e).

These electrostatic features also explain why Trp-M252 (Figs. 5b and S2c) in PbRC and D2-Trp253 (Fig. S1b) in PSII, located near Q_A_, exhibit the highest *E*_m_ values among all tryptophan residues in these reaction centers. This tendency is not unique to type-II reaction centers. It is also conserved in type-I reaction centers, such as PSI (Fig. S3). Therefore, the driving force for photoinduced electron transfer in photosynthesis arises not only from the intrinsic *E*_m_ value of each redox-active cofactor (i.e. the *E*_m_ value in the absence of the protein environment) embedded in the transmembrane region but also from protein charges primarily located in the transmembrane extrinsic regions, the ubiquitous nature of protein charges in constituting the electric field essential for facilitating electron transfer.

Interestingly, although subunit M contains only a slightly larger number of tryptophan residues (20) than subunit L (16), tryptophan residues in the transmembrane region predominantly originate from subunit M along the B-branch, with only one tryptophan from subunit L along the A-branch (Fig. 2). It is also remarkable that the contribution of protein charges to *E*_m_(Trp/Trp^•**+**^) increases along the transmembrane axis toward the cytoplasmic side (Fig. 5b). Considering this unique feature and revisiting the *E*_m_(Trp/Trp^•**+**^) values in PbRC, the tryptophan residues in subunit M, specifically, Trp-M127, M155, M271, and M268, are arranged from the spheroidene binding site toward the Q_A_ binding site in the transmembrane region, in increasing order of *E*_m_ (1190, 1223, 1285, and 1499 mV, respectively, Fig. 2).

When accounting for protein dynamics using molecular dynamics (MD) simulations, the residues exhibiting the three largest deviations in *E*_m_ values are Trp-L100 (±42 mV), Trp-M254 (±31 mV), and Trp-L25 (±30 mV), all located near the aliphatic tail of Q_A_ close to the protein surface (Fig. S4). Consistently, the PbRC crystal structure identifies two significantly different conformations of this hydrophobic tail, potentially explaining the observed fluctuations in these tryptophan residues (Fig. S5). Notably, these fluctuating resides are located outside the tryptophan residues forming the energetic cascade. Thus, the energetic cascade remains unaffected, even when considering protein dynamics in MD simulations (Fig. S4). Since tryptophan is used judiciously in proteins only where its unique properties are needed (28), the presence of this chain of tryptophan residues may play a significant role in PbRC function.

The proximity of spheroidene to the edge of this tryptophan chain suggest that spheroidene in PbRC may be part of this functional chain. The cationic state of spheroidene has been reported in the LH2 complex of *Rhodobacter sphaeroides* PbRC (51), similar to the cationic state of *β*-carotene observed in PSII (3, 16–18), although spheroidene adopts a *trans*-conformation in LH2, in contrast to its *cis*-conformation in the PbRC. In PSII, carotenoids are involved in a hole-hopping pathway to dissipate the cationic state of reaction center chlorophylls when water splitting is impaired. Furthermore, hole transfer between carotenoids and tryptophan has been experimentally observed in micelles and used to determine the *E*_m_ values of carotenoids (e.g. *β*-carotene) (52, 53). Although the *E*_m_ value for spheroidene is not reported, assuming that it is similar to *β*-carotene (∼1000 mV (52, 53)), spheroidene and the tryptophan residues in PbRC may form an electron transfer cascade, facilitating hole hopping through the tryptophan chain toward spheroidene.

**Fig. 8.**
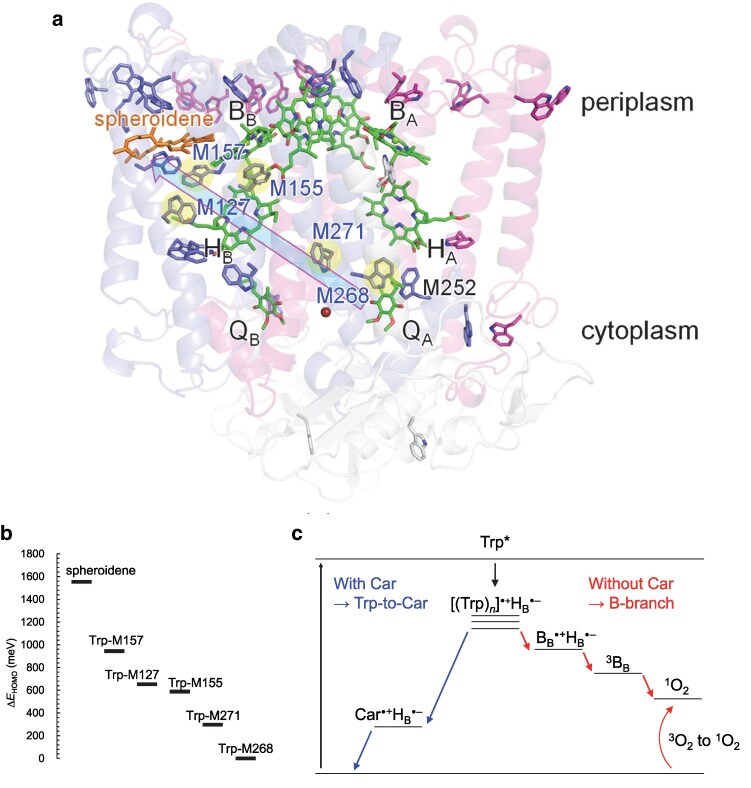
A chain of tryptophan residues from subunit M in the transmembrane region. a) Overview of the tryptophan chain. Tryptophan residues forming the chain are highlighted. b) HOMO energy levels of the tryptophan chain and spheroidene in the PbRC protein environment. The vertical axis indicates the energy difference relative to the lowest HOMO of the system, i.e. Trp-M268. c) Quenching pathways for electronically excited tryptophan in PbRC. The boxed blue arrow indicates the Trp-to-Car hole hopping pathway. The left scheme represents the pathway in the presence of spheroidene (Trp-to-Car hole hopping), while the right scheme represents the pathway in the absence of spheroidene (B-branch electron transfer).

To explore the detailed energetics of these tryptophan residues and spheroidene, QM/MM calculations are performed in the presence of the PbRC protein environment, quantum-chemically treating not only spheroidene and these tryptophan residues but also those at van der Waals contact distances from spheroidene (i.e. Trp-M66, M75, M115, M157, and M171 (27)). According to the highest occupied molecular orbital (HOMO), which correlates with the *E*_m_ value for one-electron oxidation, the following sites form an energetically downhill pathway for hole transfer: Trp-M268 → Trp-M271 → Trp-M155 → Trp-M127 → Trp-M157 → spheroidene (Car) (Fig. 8). This energy gradient is due to the electric field generated by protein charges, which facilitates electron transfer from P_A_P_B_ toward Q_A_ (Fig. 7), as removal of atomic charges from the entire PbRC protein results in the loss of this energetic cascade (Fig. S6).

These results suggest that, when one of these tryptophan residues is electronically excited upon blue or UV light absorption, the resulting hole can be transferred along this pathway to spheroidene. Notably, among the five tryptophan residues in van der Waals contact with spheroidene, the HOMO of spheroidene overlaps with Trp-M157, situated at the interface between the tryptophan chain and spheroidene (Fig. S7). This hybridization elevates the HOMO energy level of Trp-M157 and decreases its *E*_m_ value, thereby energetically integrating Trp-M157 into the tryptophan-to-carotenoid pathway (Fig. 8). This tryptophan-to-carotenoid pathway can energetically serve a photoprotective function in PbRC upon cation formation, complementing the role of spheroidene in quenching the ^3^[P_A_P_B_] triplet state via B_B_ (23).

The functional tryptophan-to-carotenoid pathway fits several experimental observations. It has been reported that while the charge separation initiates upon excitation at 860 nm, blue light irradiation (390 nm) leads to B-branch electron transfer, forming H_B_^•**−**^ without initiating from [P_A_P_B_]* (54). The blue-light-induced B-branch electron transfer, which results in a bacteriochlorophyll cation (presumably B_B_^•**+**^, not [P_A_P_B_]^•**+**^ (54)), was observed specifically in the absence of carotenoid, spheroidene (i.e. *Rhodobacter sphaeroides* strain R26, which lacks spheroidene) (54). This suggests that spheroidene inhibits blue-light-induced B-branch electron transfer in native PbRC. Thus, when the 390 nm excitation initially generates [Trp]^•^**^+^**, the absence of the tryptophan-to-carotenoid pathway in the carotenoid-less R26 PbRC eventually leads to the accumulation of B_B_^•**+**^ (*E*_m_(Trp/Trp^•**+**^) (55) > *E*_m_(BChl*a*/BChl*a*^•**+**^) (56)). The B_B_^•**+**^ state, being more stable than Trp^•**+**^, undergoes charge recombination and forms triplet B_B_, resulting in harmful ^1^O_2_ production. Indeed, in the carotenoid-less R26 PbRC, the protein was photodamaged and degraded upon UV-B irradiation (24, 54). Notably, in the H(L168)E/N(L170)D mutant PbRC, the charge separation process is pH-dependent, with a long-lived B-branch charge-separation state forming exclusively at pH 9.5 in contrast to the predominant A-branch charge-separation at pH 7.2 (57). While the *E*_m_ value of [P_A_P_B_] in this mutant is also affected by pH (57), it seems also possible that the pronounced B-branch electron transfer at high pH could be due to decreases in *E*_m_(Trp/Trp^•**+**^) (−59 mV/pH (55)), which facilitates electron release from tryptophan residues and subsequent accumulation of B_B_^•**+**^ in the PbRC.

The indole side-chain of tryptophan is the strongest UV chromophore in proteins (29), and its sacrificial absorption of UV light effectively reduced UV-induced damage to other regions of proteins (30). The cluster of tryptophan residues in the transmembrane region of the PbRC thus provides an initial layer of photoprotection. Given that the outer-sphere reorganization energy for electron transfer in PbRC is typically around 0.7 eV (e.g. H_A_ to Q_A_ (10), Q_A_ to Q_B_ (58), and bacteriochlorophylls (59)), the hole-hopping process, even when involving all tryptophan residues in the pathway, is estimated to proceed within 60 to 260 ns with outer-sphere reorganization energies of 0.6 to 0.8 eV, based on the Moser-Dutton equation (60) (Table S1). This timescale suggests that the process is sufficiently rapid for effective photoprotection. Indeed, even in the absence of carotenoid, R26 PbRC was 40 times less susceptible to activity loss and 10 times less prone to protein damage than PSII upon UV radiation (24), consistent with the greater number of tryptophan residues in the transmembrane region of PbRC relative to PSII (Figs. 2 and 3).

This protective mechanism raises an important question: if UV damage is a concern, why would nature invest in the biosynthetically costly tryptophan residues in PbRC, despite the presence of other UV-absorbing amino acids, such as tyrosine and phenylalanine? Notably, chlorin cofactors, such as bacteriochlorophylls, also absorb strongly in the UV-A and UV-B regions (26), exhibiting extinction coefficients comparable to or exceeding those of aromatic amino acids. Consequently, any UV absorption by tryptophan residues is inherently accompanied by UV absorption by bacteriochlorin cofactors in the reaction center. Among all amino acids, tryptophan stands out not only as the strongest UV chromophore in proteins (29) but also due to its lowest *E*_m_ value for one-electron oxidation (*E*_m_(Trp/Trp^•+^) = 1070 mV (55)), allowing it to participate more readily in electron transfer compared to (protonated) tyrosine (*E*_m_(Tyr-OH/Tyr-OH^•+^) = 1380 mV (55, 61)) or phenylalanine.

Given the shared UV absorption properties of bacteriochlorin and tryptophan, along with the unique redox role of tryptophan among all amino acids, the strategic positioning of tryptophan residues in PbRC serves two crucial functions: (i) preferentially absorbing UV radiation to shield the protein from direct damage and (ii) safely dissipating the resulting charge-separated state through electron hole transfer, even when tryptophan itself is photooxidized.

If the tryptophan chain were absent, continuous UV irradiation would inevitably lead to the accumulation of oxidized tryptophan photoproducts, such as *N*-formylkynurenine and kynurenine, which could further sensitize the protein to damage (29). Therefore, utilizing “hole hopping thorough tryptophan chains” from photooxidized tryptophan, as observed in proteins among all enzyme classes (33), and employing spheroidene as a terminal cation sink seem to provide a more sustainable photoprotective strategy for PbRC. Thus, if sacrificial UV absorption by tryptophan serves as the first line of defense, this hole-hopping mechanism constitutes a second line of defense, ensuring robust protection against photoinduced damage.

The absence of a corresponding tryptophan-to-carotenoid pathway in PSII may reflect an evolutionary trade-off between the high energy cost of tryptophan biosynthesis and the adoption of alternative photoprotective strategies. As tryptophan biosynthesis is the most energy-consuming pathway among all amino acids (28), minimizing the number of tryptophan residues is advantageous. Instead of relying on tryptophan, PSII employs protein degradation as a photoprotective mechanism: similar to the photodamaged D1 protein, the photodamaged D2 protein is also degraded and replaced with an intact D2 protein (20, 21). Therefore, limiting the number of energetically costly tryptophan residues in D2 and relying on protein degradation is a more economical approach for PSII. The absence of *β*-carotene near the D2-branch further supports this strategy. In contrast, PbRC, which does not evolve O_2_, is less likely to require a corresponding degradation process for subunits L and M.

The early Earth, where purple bacteria likely originated, was anaerobic, so the bacteria would not have been under evolutionary pressure to adapt to reactive oxygen species generated in oxygenic environments. In the absence of oxygen, the primary environmental stressor would have been UV radiation. In such conditions, organisms would need mechanisms to avoid UV-induced damage. Therefore, the ancestors of PbRC likely evolved to contain a high number of tryptophan residues, developing UV tolerance as a priority, allowing them to absorb and effectively dissipate harmful UV energy. This strategy involves investing resources to build a robust, long-lasting protein system.

With the evolution of ancestral PSII (e.g. (62),), capable of water splitting and O_2_ evolution, oxygen levels gradually increased on Earth. This rise in oxygen led to the formation of the ozone layer, as UV radiation converts O_2_ into O_3_. The emergence of the ozone layer reduced the exposure to harmful UV radiation, easing the selective pressure for UV protection. However, as O_2_ accumulated, photosynthetic reaction center proteins, including PSII, faced new challenges from reactive oxygen species such as ^1^O_2_.

In contrast to PbRC, PSII, having evolved later in this progressively oxygen-rich environment, developed photoprotective mechanisms centered on oxygen tolerance. Its “scrap and build” approach maintains protein function by degrading and replacing photodamaged proteins, rather than investing in a structurally robust, long-term stable framework.

Overall, PbRCs rely on UV tolerance via tryptophan, while PSII focuses on managing oxidative stress and efficiently recycling undamaged remaining protein subunits as a template for incorporating fresh, intact proteins. These divergent photoprotective strategies reflect the different environmental challenges encountered by these organisms during their emergence on Earth.

## Conclusions

The existence of an electric field in the direction of electron transfer toward Q_A_, originating from protein charges primarily located near the transmembrane extrinsic regions, is identified based on the *E*_m_ values of tryptophan residues in type-II reaction centers. The electric field is sufficiently strong to create a gradient of *E*_m_ values for tryptophan residues in the tightly packed and mostly uncharged transmembrane region, adding the driving force for electron transfer to the intrinsic *E*_m_ values of redox-active cofactors in the electron transfer branches.

Based on these findings, along with the tryptophan residues, spheroidene serves as a terminal sink for electron holes generated by photoinduced electron release from tryptophan residues in native PbRC. This mechanism would prevent the accumulation of B_B_^•**+**^ and subsequent triplet formation, thereby, preventing from harmful ^1^O_2_ generation. The absence of the functional tryptophan-to-carotenoid pathway in the carotenoid-less R26 PbRC is likely to explain why B-branch electron transfer is more pronounced (54). The presence of an electric field running along the transmembrane axis that drives electron transfer toward Q_A_, in turn, supports hole hopping in the opposite direction, terminating at spheroidene. This dual role of the transmembrane electric field serves as a fundamental design principle in photosynthetic reaction centers, maximizing light energy conversion into the driving force for electron transfer while ensuring sustainable protection against light-induced damage, thereby ultimately enhancing overall photosynthetic efficiency.

## Methods

### Coordinates and atomic partial charges

For PbRC, the initial atomic coordinates were obtained from the crystal structure of *Rhodobacter sphaeroides* PbRC (PDB code: 3I4D) (63). For PSII, the initial atomic coordinates were obtained from the X-ray structure of the PSII monomer unit (monomer A) from *Thermosynechococcus vulcanus* at a 1.9-Å resolution (PDB code: 3WU2) (64). Atomic partial charges of amino acids were derived from the all-atom CHARMM22 (65) parameter set. Atomic charges for PbRC cofactors, including bacteriochlorophyll *a*, bacteriopheophytin *a*, the nonheme Fe complex, ubiquinone, and spheroidene, and for PSII cofactors, including the Mn_4_CaO_5_ cluster (S_1_ state), chlorophyll *a*, pheophytin *a*, the nonheme Fe complex, plastoquinone, *β*-carotene, sulfoquinovosyl diacylglycerol (SQD or SQDG) were adopted from previous studies on PbRC and PSII (8). Atomic charges for digalactosyl-diacyl-glycerol (DGD or DGDG), 1,2-dipalmitoyl-phosphatidyl-glycerole (LHG or PG), and 1,2-distearoyl-monogalactosyl-diglyceride (LMG or MGDG) were adopted from previous studies on PSI (66). Atomic charges for redox-active tryptophan were obtained from studies on DNA photolyase (67). The positions of all heavy atoms were fixed, and all titratable groups, such as acidic and basic groups, were ionized during optimization of H atom positions using CHARMM (68). Ligand histidine residues were maintained in charge-neutral, singly protonated states. D1-His337 in the Mn_4_CaO_5_ cluster was protonated in PSII (69). All other acidic and basic groups were ionized. Following the established protocol (70), the resulting atomic coordinates were used, and all water molecules assigned in the crystal structure, except those serving as ligand water molecules at chlorophyll *a*, were removed (treated implicitly using a dielectric constant of 80) for subsequent *E*_m_ and QM/MM calculations.

### Protonation pattern

The protonation states of titratable residues and the redox potentials of redox-active groups were calculated by solving the linear PB equation using the MEAD program (71). To ensure consistency with previous computational results (e.g. (8, 72)), all calculations were performed at 300 K, pH 7.0, and an ionic strength of 100 mM. The dielectric constants used were 4 for the protein interior and 80 for water. p*K*_a_ values of titratable sites in the protein were determined by adding the calculated p*K*_a_ shift relative to a reference system to the known reference p*K*_a_ values: 12.0 for Arg, 4.0 for Asp, 9.5 for Cys, 4.4 for Glu, 10.4 for Lys, 9.6 for Tyr (73), and 7.0 and 6.6 for the N_ε_ and N_δ_ atoms of His, respectively (74–76). All other titratable sites were fully equilibrated to the protonation state of the target site during titration, which was performed using a Monte Carlo sampling via Karlsberg (77). The linear PB equation was solved through a three-step grid-focusing procedure at resolutions of 2.5, 1.0, and 0.3 Å. Monte Carlo sampling yielded the probabilities ([protonated] and [deprotonated]) for the two protonation states. The p*K*_a_ value was evaluated using the Henderson–Hasselbalch equation.

### *E*_m_ calculation: solving the linear PB equation

To determine *E*_m_ values in the protein, the electrostatic energy difference between two redox states in a reference model system was calculated, by solving the linear PB equation with the MEAD program (71). The experimentally measured *E*_m_(Trp/Trp^•**+**^) value of 1070 mV for one-electron oxidation (55) was used as a reference value in water. The shift in *E*_m_ value induced by the protein environment relative to the reference system was added to the known *E*_m_ value, while titrating all titratable residues. Monte Carlo sampling provided probabilities for the oxidized [*A*_ox_] and reduced [*A*_red_]) states of the molecule *A*. *E*_m_ was evaluated using the Nernst equation. A bias potential was applied to equalize the redox states ([*A*_ox_] = [*A*_red_]), yielding the redox midpoint potential as the resulting bias potential. For further discussion of the theoretical background of the PB approach, its treatment of dielectric anisotropy, and comparisons with alternative methods such as MD-based thermodynamic integration and QM/MM-based approaches, see Supporting Discussion.

### Visualization of electric field

The electric field was represented through the electrostatic potential generated by the protein complex. Using the resulting protonation pattern, the electrostatic potential was calculated by solving the Poisson equation. The dielectric constant was set to 4 for the protein and the membrane and 80 for bulk water. The equation was solved numerically within a 120 Å × 120 Å × 120 Å box comprising ∼450,000 grid points. Boundary conditions were set at 0 V for *z* = −60 Å and *z* = 60 Å. The density of the atomic partial charge was modeled as a Gaussian distribution with a standard deviation of 1 Å.

### MD simulations

To evaluate the contribution of protein dynamics from fluctuating side chains to *E*_m_(Trp) specifically in PbRC, MD simulations were conducted. The PbRC assembly was embedded in a lipid bilayer consisting of ∼200 POPC molecules and solvated with ∼16,000 water molecules. One chloride ion was added to the model using the CHARMM-GUI program (78). To ensure a direct comparison with *E*_m_(Trp) values in PSII, which were calculated based on the crystal structure, the protein backbone and cofactor groups, including their histidine and glutamate ligands of bacteriochlorophyll *a* and the nonheme Fe complex, were constrained throughout the simulations. After structural optimization with positional restraints on heavy atoms of the PbRC assembly, the system was heated from 0.1 to 300 K over 5.5 ps with a time step of 0.01 fs, equilibrated at 300 K for 0.2 ns with a time step of 0.1 fs. The final PbRC structure was obtained after equilibration at 300 K for 56 ns with a time step of 1.0 fs. All simulations were conducted using the NAMD program (79). The SHAKE algorithm (80) was applied for hydrogen constraints with a time step of 1.0 fs, where applicable. The Langevin thermostat and piston were used for temperature and pressure control, respectively (81). The *E*_m_(Trp) values were calculated using 10 conformations deduced from the final 4-ns segment of the MD simulation.

### QM/MM calculations

The unrestricted density functional theory method was employed with the B3LYP functional and LACVP* basis sets in the QSite (82) program. The QM region included spheroidene and the sidechains of Trp-M66, M75, M115, M127, M155, M157, M171, M268, and M271. All atomic coordinates were fully relaxed in the QM region. In the MM region, the positions of H atoms were optimized using the OPLS2005 force field (83), while the positions of the heavy atoms were fixed. When obtaining QM/MM-optimized geometry, the resulting protonation pattern was implemented implicitly in the titratable residues in the MM region.

The HOMO energy level of each group was calculated in the presence of the PbRC protein environment, using the QM/MM-optimized geometry, the B3LYP functional and 6-31G* basis sets, and the polarizable continuum model (PCM), as implemented in the GAMESS program (84) (QM/MM/PCM). To represent the bulk water region and explicitly account for electrostatic and steric effects from the protein environment, polarization points were placed on spheres with a 3.0 Å radius centered on each atom, and a dielectric constant of 78 was applied. See the Supporting information for the atomic coordinates of the QM/MM-optimized structures.

## Supplementary Material

pgaf278_Supplementary_Data

## Data Availability

All data is included in the manuscript and/or Supporting information.

## References

[1] Aro E-M, Virgin I, Andersson B. 1993. Photoinhibition of photosystem II. Inactivation, protein damage and turnover. Biochim Biophys Acta. 1143:113–134. 8318516 10.1016/0005-2728(93)90134-2

[2] Rutherford AW, Faller P. 2001. The heart of photosynthesis in glorious 3D. Trends Biochem Sci. 26:341–344. 11406392 10.1016/s0968-0004(01)01874-6

[3] Telfer A . 2002. What is *β*-carotene doing in the photosystem II reaction centre? Phil Trans R Soc Lond B. 357:1431–1440. 12437882 10.1098/rstb.2002.1139 PMC1693050

[4] Noguchi T . 2002. Dual role of triplet localization on the accessory chlorophyll in the photosystem II reaction center: photoprotection and photodamage of the D1 protein. Plant Cell Physiol. 43:1112–1116. 12407190 10.1093/pcp/pcf137

[5] Silva P, et al 2003. Ftsh is involved in the early stages of repair of photosystem II in *Synechosystis* sp PCC 6803. Plant Cell. 15:2152–2164. 12953117 10.1105/tpc.012609 PMC181337

[6] Tamura H, Saito K, Ishikita H. 2020. Acquirement of water-splitting ability and alteration of the charge-separation mechanism in photosynthetic reaction centers. Proc Natl Acad Sci U S A. 117:16373–16382. 32601233 10.1073/pnas.2000895117 PMC7368266

[7] Steffen MA, Lao K, Boxer SG. 1994. Dielectric asymmetry in the photosynthetic reaction center. Science. 264:810–816. 17794722 10.1126/science.264.5160.810

[8] Kawashima K, Ishikita H. 2018. Energetic insights into two electron transfer pathways in light-driven energy-converting enzymes. Chem Sci. 9:4083–4092. 29780537 10.1039/c8sc00424b PMC5944228

[9] Marcus RA, Sutin N. 1985. Electron transfers in chemistry and biology. Biochim Biophys Acta. 811:265–322.

[10] Saito K, Tamura H, Ishikita H. 2024. Superexchange electron transfer and protein matrix in the charge-separation process of photosynthetic reaction centers. J Phys Chem Lett. 15:9183–9192. 39213497 10.1021/acs.jpclett.4c02232 PMC11404480

[11] Kirmaier C, Holten D, Bylina EJ, Youvan DC. 1988. Electron transfer in a genetically modified bacterial reaction center containing a heterodimer. Proc Natl Acad Sci U S A. 85:7562–7566. 3051000 10.1073/pnas.85.20.7562 PMC282232

[12] Kirmaier C, Bautista JA, Laible PD, Hanson DK, Holten D. 2005. Probing the contribution of electronic coupling to the directionality of electron transfer in photosynthetic reaction centers. J Phys Chem B. 109:24160–24172. 16375408 10.1021/jp054726z

[13] Harris MA, et al 2013. Protein influence on charge-asymmetry of the primary donor in photosynthetic bacterial reaction centers containing a heterodimer: effects on photophysical properties and electron transfer. J Phys Chem B. 117:4028–4041. 23560569 10.1021/jp401138h

[14] Laible PD, et al 2020. Switching sides—reengineered primary charge separation in the bacterial photosynthetic reaction center. Proc Natl Acad Sci U S A. 117:865–871. 31892543 10.1073/pnas.1916119117 PMC6969525

[15] Brinkert K, De Causmaecker S, Krieger-Liszkay A, Fantuzzi A, Rutherford AW. 2016. Bicarbonate-induced redox tuning in photosystem II for regulation and protection. Proc Natl Acad Sci U S A. 113:12144–12149. 27791001 10.1073/pnas.1608862113 PMC5087022

[16] Tomo T, et al 1997. Topology of pigments in the isolated photosystem II reaction center studied by selective extraction. Biochim Biophys Acta. 1321:21–30.

[17] Faller P, Pascal A, Rutherford AW. 2001. *β*-carotene redox reactions in photosystem II: electron transfer pathway. Biochemistry. 40:6431–6440. 11371206 10.1021/bi0026021

[18] Tracewell CA, Brudvig GW. 2003. Two redox-active *β*-carotene molecule in photosystem II. Biochemistry. 42:9127–9136. 12885246 10.1021/bi0345844

[19] Kato Y, et al 2023. Characterization of tryptophan oxidation affecting D1 degradation by FtsH in the photosystem II quality control of chloroplasts. eLife. 12:RP88822. 37986577 10.7554/eLife.88822 PMC10665015

[20] Jansen MAK, Mattoo AK, Edelman M. 1999. D1-D2 protein degradation in the chloroplast. Eur J Biochem. 260:527–532. 10095791 10.1046/j.1432-1327.1999.00196.x

[21] Kale R, et al 2017. Amino acid oxidation of the D1 and D2 proteins by oxygen radicals during photoinhibition of photosystem II. Proc Natl Acad Sci U S A. 114:2988–2993. 28265052 10.1073/pnas.1618922114 PMC5358366

[22] Tandori J, Hideg É, Nagy L, Maróti P, Vass I. 2001. Photoinhibition of carotenoidless reaction centers from *Rhodobacter sphaeroides* by visible light. Effects on protein structure and electron transport. Photosynth Res. 70:175–184. 16228351 10.1023/A:1017907404325

[23] Mandal S, et al . 2017. Mechanism of triplet energy transfer in photosynthetic bacterial reaction centers. J Phys Chem B. 121:6499–6510. 28605596 10.1021/acs.jpcb.7b03373

[24] Tandori J, Máté Z, Maróti P, Vass I. 1996. Resistance of reaction centers from *Rhodobacter sphaeroides* against UV-B radiation. Effects on protein structure and electron transport. Photosynth Res. 50:171–179. 24271934 10.1007/BF00014887

[25] Nakatsuji H, Tokita Y, Hasegawa J, Hada M. 1996. Ground and excited states of carboxyheme: a SAC/SAC-CI study. Chem Phys Lett. 256:220–228.

[26] Hoff A, Amesz J. Visible absorption spectroscopy of chlorophylls. In: Chlorophylls. CRC Press, Boca Raton, 1991. p. 723–738.

[27] Roszak AW, et al 2004. Protein regulation of carotenoid binding; gatekeeper and locking amino acid residues in reaction centers of *Rhodobacter sphaeroides*. Structure. 12:765–773. 15130469 10.1016/j.str.2004.02.037

[28] Barik S . 2020. The uniqueness of tryptophan in biology: properties, metabolism, interactions and localization in proteins. Int J Mol Sci. 21:8776. 33233627 10.3390/ijms21228776 PMC7699789

[29] Pattison DI, Rahmanto AS, Davies MJ. 2012. Photo-oxidation of proteins. Photochem Photobiol Sci. 11:38–53. 21858349 10.1039/c1pp05164d

[30] Oladepo SA, Loppnow GR. 2010. The effect of tryptophan on UV-induced DNA photodamage. Photochem Photobiol. 86:844–851. 20492563 10.1111/j.1751-1097.2010.00745.x

[31] Rizzini L, et al 2011. Perception of UV-B by the *Arabidopsis* UVR8 protein. Science. 332:103–106. 21454788 10.1126/science.1200660

[32] Godik VI, Blankenship RE, Causgrove TP, Woodbury N. 1993. Time-resolved trypthophan fluorescence in photosynthetic reaction centers from *Rhodobacter sphaeroides*. FEBS Lett. 321:229–232. 8477854 10.1016/0014-5793(93)80114-a

[33] Gray HB, Winkler JR. 2015. Hole hopping through tyrosine/tryptophan chains protects proteins from oxidative damage. Proc Natl Acad Sci U S A. 112:10920–10925. 26195784 10.1073/pnas.1512704112 PMC4568215

[34] Lomize MA, Pogozheva ID, Joo H, Mosberg HI, Lomize AL. 2012. OPM database and PPM web server: resources for positioning of proteins in membranes. Nucleic Acids Res. 40:D370–D376. 21890895 10.1093/nar/gkr703 PMC3245162

[35] Saito K, Rutherford AW, Ishikita H. 2013. Mechanism of tyrosine D oxidation in photosystem II. Proc Natl Acad Sci U S A. 110:7690–7695. 23599284 10.1073/pnas.1300817110 PMC3651498

[36] Plato M, Michel-Beyerle ME, Bixon M, Jortner J. 1989. On the role of tryptophan as a superexchange mediator for quinone reduction in photosynthetic reaction centers. FEBS Lett. 249:70–74.

[37] Ito H, Nakatsuji H. 2001. Roles of proteins in the electron transfer in the photosynthetic reaction center of *Rhodopseudomonas viridis*: bacteriopheophytin to ubiquinone. J Comput Chem. 22:265–272.

[38] Saito K, et al 2012. Deformation of chlorin rings in the photosystem II crystal structure. Biochemistry. 51:4290–4299. 22568617 10.1021/bi300428s

[39] Saito K, et al 2011. Distribution of the cationic state over the chlorophyll pair of photosystem II reaction center. J Am Chem Soc. 133:14379–14388. 21805998 10.1021/ja203947k

[40] Rigby SEJ, Nugent JHA, O'Malley PJ. 1994. ENDOR and special triple resonance studies of chlorophyll cation radicals in photosystem 2. Biochemistry. 33:10043–10050. 8060973 10.1021/bi00199a031

[41] Diner BA, et al 2001. Site-directed mutations at D1-His198 and D2-His197 of photosystem II in *Synechocystis* PCC 6803: sites of primary charge separation and cation and triplet stabilization. Biochemistry. 40:9265–9281. 11478894 10.1021/bi010121r

[42] Okubo T, Tomo T, Sugiura M, Noguchi T. 2007. Perturbation of the structure of P680 and the charge distribution on its radical cation in isolated reaction center complexes of photosystem II as revealed by Fourier transform infrared spectroscopy. Biochemistry. 46:4390–4397. 17371054 10.1021/bi700157n

[43] Nakamura S, Noguchi T. 2015. Infrared detection of a proton released from tyrosine Y_D_ to the bulk upon its photo-oxidation in photosystem II. Biochemistry. 54:5045–5053. 26241205 10.1021/acs.biochem.5b00568

[44] Ishikita H, Saenger W, Loll B, Biesiadka J, Knapp E-W. 2006. Energetics of a possible proton exit pathway for water oxidation in photosystem II. Biochemistry. 45:2063–2071. 16475795 10.1021/bi051615h

[45] Rivalta I, et al 2011. Structural-functional role of chloride in photosystem II. Biochemistry. 50:6312–6315. 21678923 10.1021/bi200685w PMC3140697

[46] Debus RJ . 2014. Evidence from FTIR difference spectroscopy that D1-Asp61 influences the water reactions of the oxygen-evolving Mn_4_CaO_5_ cluster of photosystem II. Biochemistry. 53:2941–2955. 24730551 10.1021/bi500309f

[47] Narzi D, Bovi D, Guidoni L. 2014. Pathway for Mn-cluster oxidation by tyrosine-Z in the S_2_ state of photosystem II. Proc Natl Acad Sci U S A. 111:8723–8728. 24889635 10.1073/pnas.1401719111 PMC4066536

[48] Saito K, Nishio S, Ishikita H. 2023. Interplay of two low-barrier hydrogen bonds in long-distance proton-coupled electron transfer for water oxidation. PNAS Nexus. 2:pgad423. 38130665 10.1093/pnasnexus/pgad423 PMC10733176

[49] Ishikita H, Saito K. 2025. Photosystem II: probing protons and breaking barriers. Biochemistry. 64:1895–1906. 40193597 10.1021/acs.biochem.5c00112 PMC12060903

[50] Axelrod HL, et al 2002. X-ray structure determination of the cytochrome *c*_2_: reaction center electron transfer complex from *Rhodobacter sphaeroides*. J Mol Biol. 319:501–515. 12051924 10.1016/S0022-2836(02)00168-7

[51] Polívka T, et al 2002. The carotenoid S_1_ state in LH2 complexes from purple bacteria *Rhodobacter sphaeroides* and *Rhodopseudomonas acidophila*: S_1_ energies, dynamics, and carotenoid radical formation. J Phys Chem B. 106:11016–11025.

[52] Burke M, Edge R, Land EJ, McGarvey DJ, Truscott TG. 2001. One-electron reduction potentials of dietary carotenoid radical cations in aqueous micellar environments. FEBS Lett. 500:132–136. 11445071 10.1016/s0014-5793(01)02601-1

[53] Edge R, Land EJ, McGarvey DJ, Burke M, Truscott TG. 2000. The reduction potential of the *β*-carotene^•+^/*β*-carotene couple in an aqueous micro-heterogeneous environment. FEBS Lett. 471:125–127. 10767406 10.1016/s0014-5793(00)01366-1

[54] Lin S, Katilius E, Haffa ALM, Taguchi AKW, Woodbury NW. 2001. Blue light drives B-side electron transfer in bacterial photosynthetic reaction centers. Biochemistry. 40:13767–13773. 11705365 10.1021/bi015612q

[55] Tommos C, Skalicky JJ, Pilloud DL, Wand AJ, Dutton PL. 1999. De novo proteins as models of radical enzymes. Biochemistry. 38:9495–9507. 10413527 10.1021/bi990609g

[56] Watanabe T, Kobayashi M. Electrochemistry of chlorophylls. In: Scheer H, editor. Chlorophylls. CRC Press, Boca Raton, 1991. p. 287–303.

[57] Haffa ALM, et al 2004. Controlling the pathway of photosynthetic charge separation in bacterial reaction centers. J Phys Chem B. 108:4–7.

[58] Sugo Y, Tamura H, Ishikita H. 2022. Electron transfer route between quinones in type-II reaction centers. J Phys Chem B. 126:9549–9558. 36374126 10.1021/acs.jpcb.2c05713 PMC9707520

[59] Tamura H, Saito K, Ishikita H. 2021. The origin of unidirectional charge separation in photosynthetic reaction centers: nonadiabatic quantum dynamics of exciton and charge in pigment–protein complexes. Chem Sci. 12:8131–8140. 34194703 10.1039/d1sc01497h PMC8208306

[60] Page CC, Moser CC, Chen X, Dutton PL. 1999. Natural engineering principles of electron tunnelling in biological oxidation-reduction. Nature. 402:47–52. 10573417 10.1038/46972

[61] Tommos C, Babcock GT. 2000. Proton and hydrogen currents in photosynthetic water oxidation. Biochim Biophys Acta. 1458:199–219. 10812034 10.1016/s0005-2728(00)00069-4

[62] Cardona T, Murray JW, Rutherford AW. 2015. Origin and evolution of water oxidation before the last common ancestor of the cyanobacteria. Mol Biol Evol. 32:1310–1328. 25657330 10.1093/molbev/msv024 PMC4408414

[63] Roszak AW, et al 2012. New insights into the structure of the reaction centre from *Blastochloris viridis*: evolution in the laboratory. Biochem J. 442:27–37. 22054235 10.1042/BJ20111540

[64] Umena Y, Kawakami K, Shen J-R, Kamiya N. 2011. Crystal structure of oxygen-evolving photosystem II at a resolution of 1.9 Å. Nature. 473:55–60. 21499260 10.1038/nature09913

[65] MacKerell AD Jr, et al 1998. All-atom empirical potential for molecular modeling and dynamics studies of proteins. J Phys Chem B. 102:3586–3616. 24889800 10.1021/jp973084f

[66] Kawashima K, Ishikita H. 2017. Structural factors that alter the redox potential of quinones in cyanobacterial and plant photosystem I. Biochemistry. 56:3019–3028. 28530393 10.1021/acs.biochem.7b00082

[67] Popovic DM, Zmiric A, Zaric SD, Knapp E-W. 2002. Energetics of radical transfer in DNA photolyase. J Am Chem Soc. 124:3775–3782. 11929268 10.1021/ja016249d

[68] Brooks BR, et al 1983. CHARMM: a program for macromolecular energy minimization and dynamics calculations. J Comput Chem. 4:187–217.

[69] Nakamura S, Noguchi T. 2017. Infrared determination of the protonation state of a key histidine residue in the photosynthetic water oxidizing center. J Am Chem Soc. 139:9364–9375. 28635275 10.1021/jacs.7b04924

[70] Rabenstein B, Ullmann GM, Knapp E-W. 1998. Calculation of protonation patterns in proteins with structural relaxation and molecular ensembles—application to the photosynthetic reaction center. Eur Biophys J. 27:626–637.

[71] Bashford D, Karplus M. 1990. *p*K**_a_'s of ionizable groups in proteins: atomic detail from a continuum electrostatic model. Biochemistry. 29:10219–10225. 2271649 10.1021/bi00496a010

[72] Nishikawa G, Sugo Y, Saito K, Ishikita H. 2023. Absence of electron-transfer-associated changes in the time-dependent X-ray free-electron laser structures of the photosynthetic reaction center. eLife. 12:RP88955. 37796246 10.7554/eLife.88955 PMC10554733

[73] Nozaki Y, Tanford C. 1967. Acid-base titrations in concentrated guanidine hydrochloride. Dissociation constants of the guanidinium ion and of some amino acids. J Am Chem Soc. 89:736–742. 6037018 10.1021/ja00980a002

[74] Tanokura M . 1983. ^1^H nuclear magnetic resonance titration curves and microenvironments of aromatic residues in bovine pancreatic ribonuclease A. J Biochem. 94:51–62. 6619120 10.1093/oxfordjournals.jbchem.a134353

[75] Tanokura M . 1983. ^1^H-NMR study on the tautomerism of the imidazole ring of histidine residues: I. Microscopic p*K* values and molar ratios of tautomers in histidine-containing peptides. Biochim Biophys Acta. 742:576–585. 6838890 10.1016/0167-4838(83)90276-5

[76] Tanokura M . 1983. ^1^H-NMR study on the tautomerism of the imidazole ring of histidine residues: II. Microenvironments of histidine-12 and histidine-119 of bovine pancreatic ribonuclease a. Biochim Biophys Acta. 742:586–596. 6838891 10.1016/0167-4838(83)90277-7

[77] Rabenstein B, Knapp E-W. 2001. Calculated pH-dependent population and protonation of carbon-monoxy-myoglobin conformers. Biophys J. 80:1141–1150. 11222279 10.1016/S0006-3495(01)76091-2 PMC1301310

[78] Jo S, Kim T, Iyer VG, Im W. 2008. CHARMM-GUI: a web-based graphical user interface for CHARMM. J Comput Chem. 29:1859–1865. 18351591 10.1002/jcc.20945

[79] Phillips JC, et al 2005. Scalable molecular dynamics with NAMD. J Comput Chem. 26:1781–1802. 16222654 10.1002/jcc.20289 PMC2486339

[80] Ryckaert J-P, Ciccotti G, Berendsen HJC. 1977. Numerical integration of the Cartesian equations of motion of a system with constraints: molecular dynamics of *n*-alkanes. J Comput Phys. 23:327–341.

[81] Feller SE, Zhang Y, Pastor RW, Brooks BR. 1998. Constant pressure molecular dynamics simulation: The Langevin piston method. J Chem Phys. 103:4613–4621.

[82] QSite. 2012. version 5.8, Schrödinger, LLC, New York, NY.

[83] Jorgensen WL, Maxwell DS, Tirado-Rives J. 1996. Development and testing of the OPLS all-atom force field on conformational energetics and properties of organic liquids. J Am Chem Soc. 118:11225–11236.

[84] Schmidt MW, et al 1993. General atomic and molecular electronic-structure system. J Comput Chem. 14:1347–1363.

