# Supplementary material for: Energetics of tryptophan residues in electron transfer and photoprotection of type-II photosynthetic reaction centers
Source: PNAS Nexus. 2025 Sep 1;4(9):pgaf278. doi: 10.1093/pnasnexus/pgaf278 (PMC12445680; doi:10.1093/pnasnexus/pgaf278)
Supplement: pgaf278_Supplementary_Data [file pgaf278_supplementary_data.docx]

**Supporting Information**

Energetics of tryptophan residues in electron transfer and photoprotection of type-II photosynthetic reaction centers

Tomoyasu Noji ^1,2^, Keisuke Saito ^1,2^, and Hiroshi Ishikita ^1,2^*

1) Department of Applied Chemistry, The University of Tokyo, 7-3-1 Hongo, Bunkyo-ku, Tokyo 113-8654, Japan

2) Research Center for Advanced Science and Technology, The University of Tokyo, 4-6-1 Komaba, Meguro-ku, Tokyo 153-8904, Japan

CORRESPONDING AUTHOR: Ishikita, Research Center for Advanced Science and Technology, The University of Tokyo, 4-6-1 Komaba, Meguro-ku, Tokyo 153-8904, Japan, Tel. +81-3-5452-5056, Fax. +81-3-5452-5083, **E-mail:** hiro@appchem.t.u-tokyo.ac.jp

**Contents**

17 pages

1 table

7 figures

**Supporting Discussion**

**Continuum dielectric model and dielectric anisotropy in PB calculations.** The present electrostatic approach is based on the continuum dielectric model developed by Bashford and Karplus (1). This framework, or similar equivalent formulations, has long been applied to p*K*_a_ and *E*_m_ calculations for proteins by numerous research groups, including those of Gerwert (2), Gunner, Honig (3), Ullmann, and Knapp (4). In this model, the dielectric constant of water is its experimentally measured value of ~80. In contrast, the appropriate value for the “protein dielectric constant” (ε_p_), which is assigned to the protein interior (i.e., the region within the solvent-accessible surface defined by the van der Waals radii of all protein atoms), is a model-dependent parameter. As rationalized by Schutz and Warshel (5), if all physical characteristics of the protein, including electronic polarizability and conformational fluctuations, were explicitly represented, the appropriate dielectric constant would be close to 1. In practice, however, ε_p_ is assigned a higher value to implicitly account for such physical characteristics that are not explicitly modeled. In the context of this model-dependent parameter, our approach, using a value of 4 for ε_p_ best reproduce experimentally measured p*K*_a_ values and *E*_m_ values among a variety of proteins, including photosynthetic reaction centers and enzymes (e.g., (6-11)).

In our studies, while ε_p_ of 4 is used to describe the protein interior, this does not mean that the protein is treated as a spatially uniform dielectric body. The dielectric boundary between protein and solvent is defined using a solvent probe radius of 1.4 Å, capturing the molecular surface geometry, including cavity and packing feature of the protein. As a result, instead of assigning several values for ε_p_, spatial resolution is introduced, such that the actual local dielectric constant varies spatially, ranging from 4 in deeply buried regions to 80 in bulk solvent, thereby accounting for dielectric anisotropy. In addition, with the aid of Monte Carlo sampling implemented via Karlsberg (12), the protonation states of all titratable residues and the redox states of all redox-active groups are electrostatically coupled and treated in thermodynamic equilibrium. This captures additional configurational and electrostatic features that cannot be represented simply by variations in dielectric constants, further enhancing the model’s ability to account for dielectric anisotropy. These combined features ensure that the anisotropic nature of the protein environment is effectively represented in the present PB-based framework.

**Comparisons with other approaches for *E*_m_ calculations.** Because *E*_m_ and p*K*_a_ values of titratable groups in protein environments are predominantly determined by the cumulative electrostatic interactions with surrounding charges, solving the linear PB equation provides the most direct, electrostatics-based approach for calculating *E*_m_ values, grounded in the physical origin of *E*_m_ shifts in protein environments.

Alternatively, molecular dynamics (MD)-based thermodynamic integration (TI) was previously applied to estimate the *E*_m_ values of a multiheme protein, MtrF (13). However, due to the nature of MD simulations, it requires fixing the protonation states of all titratable groups, including the heme propionates, throughout the simulations. This is problematic, as the protonation states of heme propionates are strongly coupled to the heme redox state and critically affects the calculated *E*_m_ values (14). Consequently, the MD-based TI approach required uniform empirical shifts of –1567 mV to match the calculated *E*_m_ values with experimentally measured *E*_m_ ranges, thereby obscuring the physical origins of the electrostatic contributions.

In contrast, our PB-based approach allows the protonation states of all titratable groups, including heme propionates, to vary dynamically in response to changes in the heme redox state (15). This enables us to reproduce *E*_m_ values without empirical adjustment, using only the experimentally determined *E*_m_ value of a bis-histidine ligated heme in water (16) as a reference. Similarly, the MD-based TI approach also produced unphysically large electrostatic contributions from individual residues (e.g., –2280 mV shift from Asp228 (13)). Such extreme shifts have never been observed experimentally in mutagenesis studies of heme proteins, further highlighting the limitations and immaturity of the MD-based TI approach for *E*_m_ calculations (17) (note: –61 mV shift from Asp228 in the PB-based approach (15)). Taken together, the PB framework, with appropriate treatment of protonation equilibria and an empirically supported ε_p_ of 4, is a more robust and established method for calculating relevant *E*_m_ values in proteins.

On the other hand, *E*_m_ values, corresponding to one-electron oxidation or reduction, are directly related to the energy levels of the HOMO or lowest unoccupied (LUMO) molecular orbitals, respectively. Therefore, *E*_m_ values can also be reliably calculated using QM/MM-based approaches, provided that the protonation states of titratable groups are appropriately assigned in advance in the surrounding protein electrostatic environment (i.e., the MM region) (18). In general, as long as (i) redox-active groups are not quantum-mechanically coupled with adjacent groups and (ii) the *E*_m_ value of the isolated redox-active site in solvent is reported, both PB-based (e.g., (10))) and QM/MM-based (e.g., (19)) approaches yield consistent results. However, (i) when redox-active groups are quantum-mechanically coupled with neighboring groups, as in the case of the electronically coupled bacteriochlorophyll pair (P_A_ and P_B_) (19), or (ii) when the *E*_m_ value of the isolated redox site in solvent is not experimentally available, as in the case of the Mn_4_CaO_5_ cluster in photosystem II (18), QM/MM-based approaches become the only feasible method to access the *E*_m_ value in the protein environment.

**Supporting References**

1. D. Bashford, M. Karplus, pKa's of ionizable groups in proteins: atomic detail from a continuum electrostatic model. *Biochemistry* **29**, 10219-10225 (1990).

2. D. Bashford, K. Gerwert, Electrostatic calculations of the p*K*_a_ values of ionizable groups in bacteriorhodopsin. *J Mol Biol* **224**, 473-486 (1992).

3. M. R. Gunner, A. Nicholls, B. Honig, Electrostatic potentials in *Rhodopseudomonas viridis* reaction centers: implications for the driving force and directionality of electron transfer. *J. Phys. Chem.* **100**, 4277-4291 (1996).

4. G. M. Ullmann, E.-W. Knapp, Electrostatic models for computing protonation and redox equilibria in proteins. *Eur. Bophys. J.* **28**, 533-551 (1999).

5. C. N. Schutz, A. Warshel, What are the dielectric constants of proteins and how to validate electrostatic models? *Proteins* **44**, 400-417 (2001).

6. H. Ishikita, E.-W. Knapp, Variation of Ser-L223 hydrogen bonding with the Q_B_ redox state in reaction centers from *Rhodobacter sphaeroides*. *J. Am. Chem. Soc.* **126**, 8059-8064 (2004).

7. H. Ishikita, J. Biesiadka, B. Loll, W. Saenger, E.-W. Knapp, Cationic state of accessory chlorophyll and electron transfer through pheophytin to plastoquinone in photosystem II. *Angew. Chem. Int. Ed.* **45**, 1964-1965 (2006).

8. H. Ishikita, Origin of the p*K*_a_ shift of the catalytic lysine in acetoacetate decarboxylase. *FEBS Lett* **584**, 3464-3468 (2010).

9. H. Ishikita, B. T. Eger, K. Okamoto, T. Nishino, E. F. Pai, Protein conformational gating of enzymatic activity in xanthine oxidoreductase. *J Am Chem Soc* **134**, 999-1009 (2012).

10. K. Kawashima, H. Ishikita, Energetic insights into two electron transfer pathways in light-driven energy-converting enzymes. *Chem Sci* **9**, 4083-4092 (2018).

11. H. Ishikita, W. Saenger, J. Biesiadka, B. Loll, E.-W. Knapp, How photosynthetic reaction centers control oxidation power in chlorophyll pairs P680, P700, and P870. *Proc. Natl. Acad. Sci. USA* **103**, 9855-9860 (2006).

12. B. Rabenstein, E.-W. Knapp, Calculated pH-dependent population and protonation of carbon-monoxy-myoglobin conformers. *Biophysical Journal* **80**, 1141-1150 (2001).

13. M. Breuer, P. Zarzycki, J. Blumberger, K. M. Rosso, Thermodynamics of electron flow in the bacterial deca-heme cytochrome MtrF. *J Am Chem Soc* **134**, 9868-9871 (2012).

14. H. Ishikita, E.-W. Knapp, Redox potential of cytochrome *c*550 in the cyanobacterium *Thermosynechococcus elongatus*. *FEBS Lett.* **579**, 3190-3194 (2005).

15. H. C. Watanabe, Y. Yamashita, H. Ishikita, Electron transfer pathways in a multiheme cytochrome MtrF. *Proc Natl Acad Sci U S A* **114**, 2916-2921 (2017).

16. G. S. Wilson, Electrochemical studies of porphyrin redox reactions as cytochrome models. *Bioelectrochem. Bioenerg.* **1**, 172-179 (1983).

17. H. C. Watanabe, Y. Yamashita, H. Ishikita, Reply to Breuer et al.: Molecular dynamics simulations do not provide functionally relevant values of redox potential in MtrF. *Proc Natl Acad Sci U S A* **114**, E10029-E10030 (2017).

18. M. Mandal, K. Kawashima, K. Saito, H. Ishikita, Redox potential of the oxygen-evolving complex in the electron transfer cascade of photosystem II. *J. Phys. Chem. Lett.* **11**, 249-255 (2020).

19. H. Tamura, K. Saito, H. Ishikita, Acquirement of water-splitting ability and alteration of the charge-separation mechanism in photosynthetic reaction centers. *Proc. Natl. Acad. Sci. U. S. A.* **117**, 16373-16382 (2020).

**Table S1.** Estimated hole hopping rate, τ_et_. λ: outer-sphere reorganization energy.

| **donor** | **acceptor** | **distance (Å)** | ***E*_m_ (mV)** |  | **τ_et_** |  |  |
| --- | --- | --- | --- | --- | --- | --- | --- |
|  |  |  | **donor** | **acceptor** | **λ: 0.6 eV** | **0.7 eV** | **0.8 eV** |
| Trp-M268 | Trp-M271 | 6.3 | 1499 | 1285 | 1.7 ns | 3.2 ns | 6.1 ns |
| Trp-M271 | Trp-M155 | 10.9 | 1285 | 1223 | 62.2 ns | 126.2 ns | 256.5 ns |
| Trp-M155 | Trp-M127 | 7.9 | 1223 | 1190 | 0.6 ns | 1.2 ns | 2.5 ns |
|  | Trp-M157 | 7.8 | 1223 | 1297 | 0.1 ns | 0.3 ns | 0.5 ns |
| Trp-M127 | Trp-M157 | 3.8 | 1190 | 1297 | 0.4 ps | 0.7 ps | 1.4 ps |


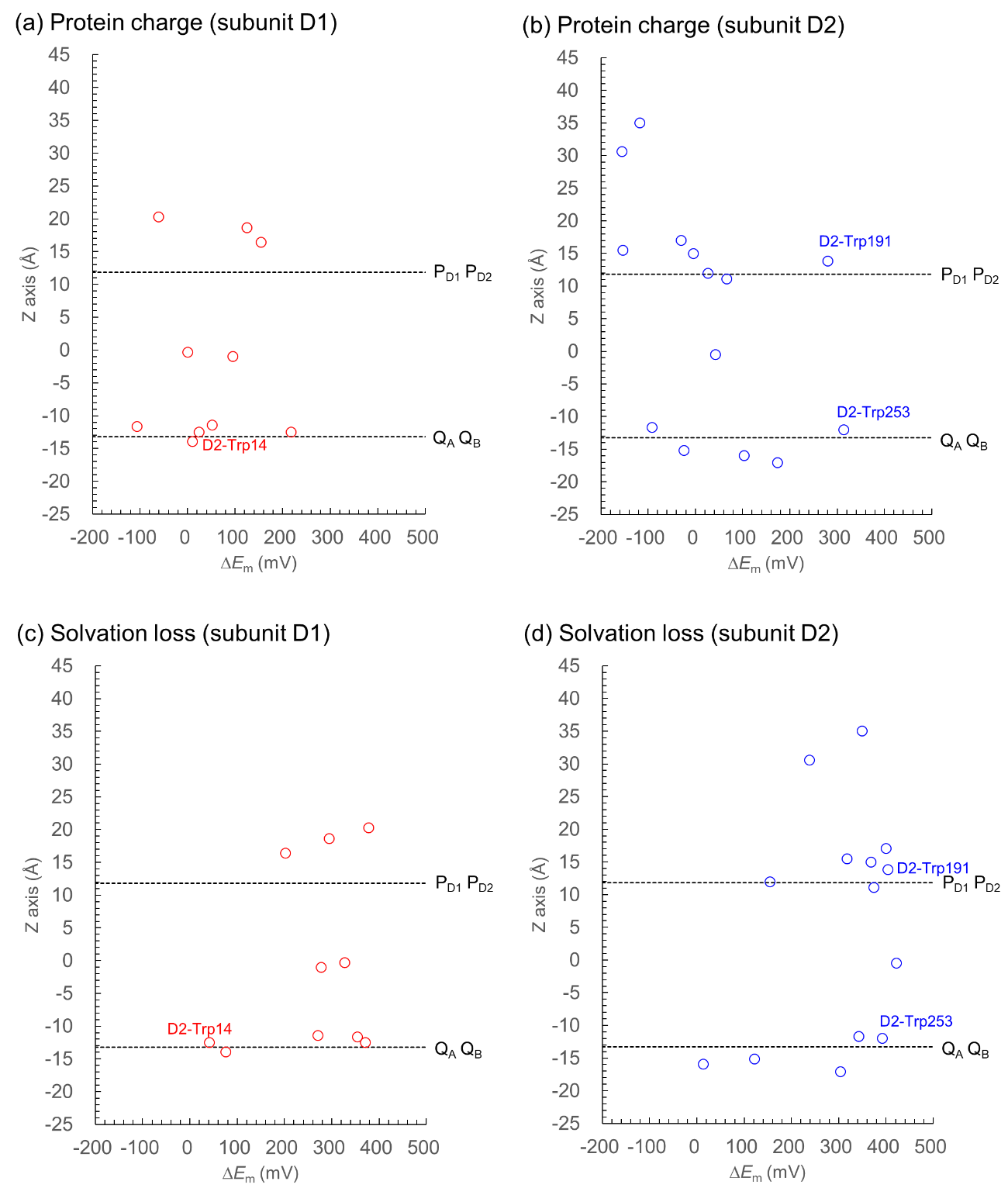


**Figure S1.** Contributions of the PSII protein environment to shifts in *E*_m_(Trp/Trp^•^**^+^**) (Δ*E*_m_). (a) Contributions of protein charges to shifts in *E*_m_(Trp/Trp^•^**^+^**) in tryptophan residues of D1 protein. (b) Contributions of protein charges to shifts in *E*_m_(Trp/Trp^•^**^+^**) in tryptophan residues of D2 protein. (c) Contributions of solvation loss to shifts in *E*_m_(Trp/Trp^•^**^+^**) in tryptophan residues of D1 protein. (d) Contributions of solvation loss to shifts in *E*_m_(Trp/Trp^•^**^+^**) in tryptophan residues of D2 protein. Red and blue open circles indicate shifts in *E*_m_(Trp/Trp^•^**^+^**) in tryptophan residues of D1 and D2 proteins, respectively. Dotted horizontal lines represent the positions of P_D1_P_D2_ and quinones, i.e., the transmembrane region.

**Figure S2.** Electric field generated by protein charges in the PbRC components. (a) Electric field generated by the membrane intrinsic region of the entire PbRC protein. (b) Electric field generated by the membrane extrinsic region of the entire PbRC protein. (c) Electric field generated by protein subunit L. (d) Electric field generated by protein subunit M. (e) Electric field generated by protein subunit H. (f) Electric field generated by the protein subunit M. (e) Electric field generated by the cofactor groups, including bacteriochlorophyll, bacteriopheophytin, the non-heme Fe complex, ubiquinone, and sphaeroidene.

**Figure S3.** Localization of negative and positive charges along the transmembrane axis in PSI with subunits PsaA and PsaB.

**Figure S4.** Deviation of *E*_m_(Trp/Trp^•^**^+^**) values in subunits L and M of the PbRC structure during MD simulations. Black horizontal bars indicate standard deviations. The transmembrane Z-axis is defined based on the OPM database. The gray horizontal line on the periplasm side represents the interface between the transmembrane and bulk regions, as defined based on the OPM database. The corresponding interface on the cytoplasm side aligns closely with the positions of quinones and is not shown for clarity. Red and blue circles indicate *E*_m_ values in subunits L and M, respectively. Dotted horizontal lines represent the positions of cofactors along the transmembrane Z-axis. Labels are provided only for residues involved in the Trp-to-Car hole hopping pathway for clarity. Residues exhibiting the three largest deviations, Trp-L100 (±42 mV), Trp-M254 (±31 mV), and Trp-L25 (±30 mV), are indicated in parentheses.

**Figure S5.** Two distinct conformations of the aliphatic tail of Q_A_ in proximity to the three highly fluctuating tryptophan residues, Trp-L100, Trp-M254, and Trp-L25, observed in the PbRC crystal structure (PDB code: 3I4D).

**Figure S6.** HOMO energy levels of the tryptophan chain and spheroidene calculated in the absence of atomic charges from the entire PbRC protein, which is the source of the electric field.

**Figure S7.** Delocalization of the spheroidene HOMO over tryptophan residues within van der Waals contact distances. The HOMO predominantly extends over Trp-M157. While the full PbRC protein environment is included electrostatically in the QM/MM calculations, only the tryptophan residues within van der Waals contact distances of spheroidene, treated quantum-chemically, are shown for clarity.
